# Research note: Impacts of piling behavior on temperature and carbon dioxide in laying hen sheds

**DOI:** 10.1016/j.psj.2024.104672

**Published:** 2024-12-17

**Authors:** Helen E. Gray, Jack O'Sullivan, Lucy Asher

**Affiliations:** School of Natural and Environmental Sciences, Newcastle University, NE1 7RU, UK

**Keywords:** Smothering, Piling, Heat stress, Environmental stressor, Greenhouse gas

## Abstract

Piling, a high density of chickens choosing to gather, is increasingly being recognized as a major problem behavior in the laying hen industry with both economic and welfare impacts. Groups of animals in close proximity generate heat, and observations of piling have noted instances of over 1200 hens in direct contact. Here, we investigate the impact of piling behavior on the temperature of the chicken shed. Since heat stress causes panting in chickens, piling also has potential to increase the CO_2_ concentration and as such, we also investigated the impact of piling on CO_2_. We used annotations of piling behavior from video footage of approximately 21 days for each of 12 flocks. The Birdbox system for flock management was used to obtain matched logged temperature (°C) and CO_2_ (ppm) from two sensor stations every minute, resulting in 17,396 datapoints. Bayesian multilevel modelling was used to estimate the effects of pile number and duration on temperature and CO_2_, including an effect to control for time of day. Since baseline daily fluctuations in temperature and CO_2_ could not be obtained, time of day effects were modelled in different ways, as autoregressive, random intercept, sinusoidal and polynomial terms. As autoregressive and non-autoregressive models could not be directly compared, we present the results of the autoregressive and best fit non-autoregressive models. We found no association between piling and temperature or CO_2_ for the autoregressive models but did find an association between pile number, pile duration and temperature in the random effects model. Higher temperature was associated with an interaction between increasing pile numbers and increasing pile duration. Since the effect size was very small and this result was not replicated in the autoregression model it should be interpreted with caution but does provide interesting rationale for future work investigating behavior-environment interactions.

## Introduction

Recurrent piling behavior refers to the crowding of laying hens into dense groups (Gray et al., 2020). When this escalates to mortality, it is commonly referred to as “smothering”. In comparison with other laying hen maladaptive behaviors (such as feather pecking), there is very little published literature addressing the causes and consequences of piling. From the few published papers available and from our own observations (e.g. Armstrong et al., 2023), we typically see piling as a slow-moving, vortex-like behavior in UK, brown hen flocks, which can occur across the day but is prevalent in the afternoon. In European white hybrids, the behavior presents differently, with groups of birds aligned uniformly and mostly immobile (Winter et al., 2021). Regardless of the form the behavior takes, the congregation of large groups of birds in a small space makes this a risky behavior for hen health and welfare.

Chowdhury et al. (2024) found smothering to account for 9-22 % of mortality in 84 Australian flocks. Most smothering events led to small numbers of deaths (median of 10), but mortality of a single event reached up to 900 birds. The scale of the problem highlights that smothering is both a welfare concern and an economic threat to egg producers. It is therefore imperative that we better understand the causes and consequences.

Previous studies report producers’ beliefs that smothering is linked to changes in temperature, for instance that sunny days result in smothers (Rayner et al., 2016). In a hypothesis paper, we argued a slightly different association: given the high numbers and density of birds, piling may be a cause of heat stress, rather than hot temperatures triggering piling (Gray et al., 2020). For animals in close contact, metabolic heat can transfer from one individual to another and radiating heat has less space to escape. Groups of animals in contact can raise the immediate temperature considerably, e.g. emperor penguin huddles maintain temperatures between 20 to 37.5°C in -15°C conditions (Gilbert et al., 2006). As piling can result in densities of up to 187.93 birds/m^2^ (Herbert et al., 2021), there is great potential for heat generation within a pile.

Heat stress in laying hens is well studied and results in wing spreading and panting, physiological changes in blood pH, reduced immune function, and impacts on egg production (Vandana et al., 2021). Though UK sheds have ventilation systems to attempt maintenance of a consistent temperature range, extreme weather events present an ever-increasing challenge in keeping animal housing cool. If piling behavior is exacerbating the temperature experienced by laying hens, it is important to understand the degree to which this is occurring.

Secondary to heat stress, panting, exhibited by birds to cool themselves, may potentially increase the concentration of CO_2_ in the shed. Carbon dioxide exposure is not a known welfare concern for laying hens unless experienced at values far above those in a shed. Anderson et al. (1966) studied the effects of 1500ppm and 5000ppm CO_2_ on feed intake, water intake and weight gain, as well as pathological changes in the respiratory tract. No differences were found in those chickens exposed to CO_2_ versus a control group, suggesting CO_2_ was not damaging at the level studied.

Though CO_2_ does not pose a known welfare concern, exploring the effects of piling on CO_2_ may be useful for understanding associations between animal behavior and greenhouse gas levels, and for detecting piling events. Our previous work indicates that piling behavior is more common than first thought. We found piling to be present in twelve monitored flocks, even when there was no known problem (Armstrong et al., 2023). The detection of piling, therefore, may be aided by understanding the changes in environmental conditions associated with the behavior.

We used sensor data from laying hen sheds to investigate the impact of piling on environmental temperature and carbon dioxide, with the following specific hypotheses:

1. An increase in piling behavior will be associated with an increase of temperature in the shed.
2. An increase in piling behavior will be associated with an increase in the concentration of CO_2_ in the shed.

## Materials and methods

This study was approved by the Newcastle University Animal Welfare Ethical Review Board (Ref: 839). We followed an open science approach, pre-registering our hypotheses and methods (https://doi.org/10.17605/OSF.IO/HD842).

The details pertaining to the observation and quantification of piling behavior have been previously described in detail (Armstrong et al., 2023). Briefly, piling behavior was described for 12 flocks located on 6 farms, in Cumbria (Northwest England). Flocks were between ∼3000-16,000 birds and filmed from overhead to capture focal portions of the litter (i.e. not all litter covered). The number and duration of piles were quantified using manual annotations over a period of approximately 21 days for each flock (see Table 1), using the following definition: *At least 30 birds aggregated in the closest possible proximity (overlapping body outlines) for at least 1 min while performing no other discernible behaviors (e.g., dustbathing), but they may be moving.*

**Table 1:** A per-flock overview of the number of data points analysed, the dates relating to data collection period, and the median temperature (°C) and CO_2_ (ppm) averaged over the recorded period (start to end date) from 8am-8pm.

Flock	Breed	No. of data points	Start date	End date	Median temp.	Median CO2	Start age
Flock 1	Shaver Brown	1584	25/10/21	15/11/21	18.76	1162.5	59
Flock 2	Shaver Brown	1584	13/10/21	03/11/21	18.55	515.5	57
Flock 3	Shaver Brown	1482	13/10/21	03/11/21	18.40	881.25	57
Flock 4	Lohmann Classic	1512	25/07/22	14/08/22	26.74	936	19
Flock 5	Shaver Brown	1506	06/07/22	27/07/22	24.95	845	21
Flock 6	Shaver Brown	1434	06/07/22	28/07/22	24.64	905.25	21
Flock 7	Shaver Brown	1656	06/07/22	28/07/22	24.64	916	18
Flock 8	Shaver Brown	1656	06/07/22	28/07/22	25.10	905	18
Flock 9	Shaver Brown	1152	05/01/22	25/01/22	21.82	2151.25	72
Flock 10	Shaver Brown	1458	16/05/22	05/06/22	25.25	1569	28
Flock 11	Shaver Brown	1512	26/08/22	15/09/22	26.18	1080.75	59
Flock 12	Lohmann Classic	1512	15/03/22	04/04/22	20.25	1320.5	30

Temperature and CO_2_ data matched to the observation period of each flock were acquired from the shed flock management system (BirdBox; FAI). Each shed had two sensor stations that logged temperature (°C) and CO_2_ (ppm) every minute. Sensor placement was shed-dependent but generally, at install, sensors were placed on either side of the house over the litter area and positioned at one-third the length of the house and two-thirds the length of the house. Sensors are positioned over the slats, approximately 2m laterally and 1.5m vertically away from the edge of the litter. Note that sensors were part of routine flock management and used opportunistically in this study.

All data were prepared for analysis using R via RStudio (v4.3.2). The annotated data periods were subdivided into ten-minute windows, with the number of separate piling events occurring providing a total piling count per window. Where piling occurred across windows the event was counted for each. A total of 3,820 observation windows contained at least 1 occurrence of piling with a mean of 1.5 occurrences within these windows.

Environmental data comprised 675,965 observations, each containing the temperature and/or CO_2_ values collected per minute by one of a shed's two sensor stations. In the case that one or both sensors had not recorded, the observation contained an NA. To pair these with the windowed observation data required a series of data cleaning steps.

1. Data outside of the 8am to 8pm observation periods were removed, resulting in 338,310 observations.
2. Outliers were identified and removed. Outliers were defined as any reading falling outside of the BirdBox's pre-selected alert extremes. The outlier thresholds for temperature were a minimum of 5°C and maximum of 37°C, and for CO_2_ any value exceeding 5,000ppm. 608 temperature outliers were removed (all > 37°C) and no outliers were identified for CO_2_.
3. Data were separated into ten-minute windows, with start and end times aligning with observation data. Data from both sensor stations were included within appropriate windows to better capture the diversity of environmental features across each shed.
4. Medians and upper quartiles of temperature and CO_2_ were calculated for each ten-minute window. Median and upper quartile were selected to reduce the influence of any extreme values in averaging.
5. Environmental windowed data were compared to a list of camera malfunction periods and those occurring during these times were removed, resulting in 850 windows of environmental data that were omitted.
6. Observation data and summarised environmental data were bound together by matching timestamps for statistical analysis. The combined dataset comprised 17,396 10-minute windows, of which 3,820 (21.96 %) contained at least one piling event.

### Statistical methods

All analyses and data visualization were conducted using R via RStudio (v4.3.2). Models were computed using the Stan programming language via the brms package (version 2.20.4), which estimates parameters using Hamiltonian Monte Carlo. Four Markov chains were run, each with a warm-up period of 2,500 iterations and 5,000 iterations used for sampling. Thinning was set to 1. Convergence was checked using the Gelman-Rubin statistic with convergence indicated by values close to 1 and less than 1.05. The R code specifying the models is available at DOI 10.17605/OSF.IO/5VQ2G.

We present the model equations below as programming syntax can change over time and by including the theoretical basis, we hope to improve the reproducibility of our results.

Given that our previous work has shown piling to be widespread amongst laying hen flocks, we could not use historic environmental data to model baseline daily fluctuations in temperature and CO_2_ as piling may have impacted these values with no way to assess this. We therefore chose to include time in our models in four different ways and then evaluate the model fits for both temperature and CO_2_.

The temperature intercept was given a normally distributed prior with a mean of 20 and SD of 5, i.e. (normal (20,5)). The CO_2_ intercept was given a normal (0,5) prior. For the autoregression models, phi (ϕ), the parameter pertaining to the correlation between timepoints was given a normal (0,1) prior with a lower bound of -1 and an upper bound of 1. Beta coefficients of piling variables were given normal (0,1) priors. Prior predictive checks were conducted using the brms package to check that the assigned priors resulted in sensible intercept estimates.

### Model 1: Random effect of time

$$E_{fht}∼PDF\left(\mu_{fht},\sigma \right)$$

$$\mu_{fht}\;=\;\alpha_{1}\;+\;\left(\beta_{1\;}+\;\tau_{1_{f}}\right)\cdot N_{fht}\;+\;\;\left(\beta_{2\;}+\;\tau_{2_{f}}\right)\cdot T_{fht}+\;\beta_{3}N_{fht}\cdot T_{fht}+\;\tau_{3_{f}}+\delta_{1_{h}}$$

Whereby, the environmental variable of interest (E, temperature or concentration of carbon dioxide) in the shed of a flock (f) at a given hour (h), for time (t) has mean ($\mu_{fht}$) and standard deviation (σ) from a suitable probability density function (PDF), which was Gaussian for temperature and lognormal for CO_2_. The mean is a function of the intercept ($\alpha_{1}$), the number of piling events with a random slope per flock ($\;\left(\beta_{1\;}+\;\tau_{1_{f}}\right)\cdot N_{fht}$), the time since an absence of piling with a random slope per flock ($\;\left(\beta_{2\;}+\;\tau_{2_{f}}\right)\cdot T_{fht}$) an interaction between the number of piles and the time since piling absence ($\beta_{3}N_{fht}\cdot T_{fht}$) and crossed random intercepts of the flock ($\tau_{3_{f}}$) and hour of day ($\delta_{1_{h}}$).

### Model 2: Autoregression of time

$$E_{ft}∼PDF\left(\mu_{ft},\sigma \right)$$

$$\mu_{ft}\;=\;\left(1\;-\;\phi \right)\alpha \;+\;\phi E_{f\left[t-1\right]}\;+\;\left(\beta_{1\;}+\;\tau_{1_{f}}\right)\cdot N_{ft}\;+\;\;\left(\beta_{2\;}+\;\tau_{2_{f}}\right)\cdot T_{ft}+\;\beta_{3}N_{ft}\cdot T_{ft}+\tau_{3_{f}}$$

Model 2 is as Model 1, but with the inclusion of parameter phi (ϕ) which represents an autoregressive component. The intercept is now defined as ((1−ϕ)α) and the random intercept of the hour of day ($\delta_{1_{h}}$) is replaced by a predictor of the environmental variable of interest at the previous time point ($\phi E_{f[t-1]}$). When phi is 1, the time series is fully impacted by the previous environmental value, when phi is zero the time series reverts to the alpha intercept.

### Model 3: Sinusoidal function of time

$$E_{ft}∼PDF\left(\mu_{ft},\sigma \right)$$

$$\mu_{ft}=\;\alpha_{1}\;+\;\left(\beta_{1\;}+\;\tau_{1_{f}}\right)\cdot N_{ft}\;+\;\;\left(\beta_{2\;}+\;\tau_{2_{f}}\right).T_{ft}+\;\beta_{3}N_{ft}\cdot T_{ft}+\beta_{4}cos\left(\frac{2\pi H}{12}\right)+\beta_{5}sin\left(\frac{2\pi H}{12}\right)+\tau_{3_{f}}$$

Model 3 is as Model 1, but the random intercept of the hour of day ($\delta_{1_{h}}$) is replaced by ($\beta_{4}cos\left(\frac{2\pi H}{12}\right)+\beta_{5}sin\left(\frac{2\pi H}{12}\right)$) which models the hour of day (H) as a sinusoidal function.

### Model 4: Polynomial function of time

$$E_{ft}∼PDF\left(\mu_{ft},\sigma \right)$$

$$\mu_{ft}=\;\alpha_{1}\;+\;\left(\beta_{1\;}+\;\tau_{1_{f}}\right)\cdot N_{ft}\;+\;\;\left(\beta_{2\;}+\;\tau_{2_{f}}\right)\cdot T_{ft}+\;\beta_{3}N_{ft}\cdot T_{ft}+\beta_{4}H_{ft}+\;\beta_{5}H_{ft}^{2}+\tau_{3_{f}}$$

Model 4 is as Model 1, but the random intercept of the hour of day ($\delta_{1_{h}}$) is replaced by ($\beta_{4}H_{ft}+\;\beta_{5}H_{ft}^{2}$)which models the hour of day (H) as a polynomial function.

#### Model fit

For each response variable, model comparisons were conducted using leave one out (LOO) cross validation (in the brms package), whereby the model with lowest information criteria score (defined as negative 2 times the expected log predictive density) indicated the best fit.

## Results and discussion

The final dataset contained 17,396 datapoints spread across 12 flocks. The medians of the raw environmental data are shown for each flock in Table 1. All results below are model estimates reported to three significant figures, unless more precision is required to accurately portray the result.

### Model fit

Model 2 was the best fit for temperature (LOO information criteria: model 1 = 82667; model 2 = -17,226; model 3 = 82982; model 4 = 83996). This indicates that temperature is best modelled temporally using an autoregression parameter. Model 2 was also the best fit for CO_2_ (LOO information criteria: model 1 = 66201; model 2 = 41228; model 3 = 66693; model 4 = 66386).

However, the LOO method of assessing model fit is not directly comparable between model 2 and models 1, 3 and 4. This is because autoregression models have high serial correlation (dependence on previous timepoints) and are non-stationary (there is a time-based trend in the data). There is the option with time series data to use a leave forward out (LFO) method of assessing model fit. However, this is not yet readily implementable in the software used in the current study.

As the models are not directly comparable, we have chosen to present both the results of the autoregression model, and the next best fitting model (random intercept). We explore the implications of modelling time differently in the discussion.

### Temperature

#### Autoregressive model

Temperature was estimated at an average of 27.6°C (95 % HDI: 23.4, 31.8). The temperature at one timepoint was strongly associated with the temperature at the previous timepoint (ϕ estimate: 0.9957; 95 % HDI: 0.9949, 0.9965). The maximum value that ϕ can take is 1, which would signify a random walk, indicating that the current value is equal to the previous, plus some error. There was no significant interaction (estimate: 8.19e-06; 95 % HDI: -0.00079, 0.000773) nor main effects of the number of piling events (estimate: 0.00312; 95 % HDI: -0.00378, 0.0102) or piling duration (estimate: 7.92e-05; 95 % HDI: -0.00262, 0.00266).

#### Random intercept model

**Figure 1:**
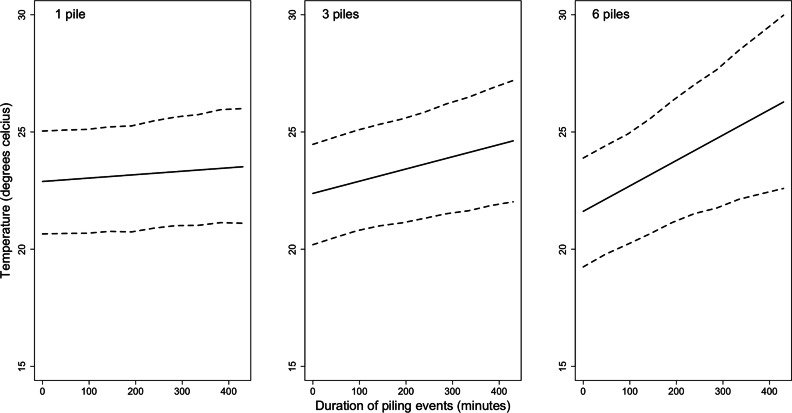
The interaction between the number and duration of piling events and the temperature, as estimated by a mixed effects model. The posterior mean is denoted by the solid black line with the 95 % highest density interval of the mean shown by the dashed lines.

The next best fitting model after autoregression, included time as a random intercept. This model estimated average temperature as 23.1°C (95 % HDI: 20.9, 25.5). There was a significant interaction (estimate: 0.0188; 95 % HDI: 0.00418, 0.0323) As the piling duration increased, temperature increased, with the effect being more pronounced in the presence of higher numbers of piles (Fig. 1). To examine two relevant examples: With one pile present, the estimated increase in temperature from 10 minutes piling duration to 50 minutes (the median) duration was 0.06°C. For six piles (the maximum we observed at one time), the estimated increase in temperature from 10 minutes to 50 minutes piling duration was 0.43°C.

We highlight that the intercept estimates are different for these two models. In the autoregressive time series model, the intercept relates to the long-term mean (i.e. where the time series reaches equilibrium). In the random intercept model, the intercept is closer to the arithmetic mean (the average of the temperature in that day).

The temperature results are promising from a welfare perspective, in that piling behavior is not causing large temperature changes in the shed, though the evidence of an interaction of pile numbers and cumulative duration is interesting. However, the sensors in this study were installed for flock management purposes and located at height above the litter. It could be that the temperature experienced within a piling event may be magnitudes different than those measured more broadly in the shed, or that proximity of a pile from a sensor affects the detection of change. Additionally, the inclusion of relative humidity levels would further aid our understanding, as humidity affects experienced temperature. Humidity sensors were fitted in the sheds, however, gas sensor responsiveness can degrade over time, and, in some cases, sensor accuracy was lower than required for study purposes. As a result, humidity readings were excluded from the analysis. Combining temperature and humidity together provides a single representative value called the temperature-humidity index (THI). The effect of varying temperature-humidity index values has been studied in the Hy-line brown strain, for panting and mortality (Kang et al., 2020). The authors found that the speed of increase in the temperature-humidity index had significant outcomes on mortality, with a more acute increase resulting in higher mortality. Panting was consistently high at a THI value equivalent to 30°C but was more variable under 30°C. For species kept in large numbers such as laying hens, linking tangible behavioral measures of heat stress to environmentally measurable variables is valuable. Directly measuring panting is challenging, however, mapping thresholds of THI which induce panting (for the relevant strain/hybrid) onto an alert system could be used to indicate heat stress during piling or other events. Therefore, though we observed small increases in shed temperature with piling events, we believe future work should include humidity and temperature measures at bird height, or using animal-mounted wearable sensors, to better understand the thermal experiences of chickens in a pile.

### CO_2_

#### Autoregressive model

The autoregression model estimated CO_2_ to be an average of 1020 (95 % HDI: 777, 1264). As with temperature, we found that CO_2_ also had a strong autocorrelated component, with measures at one timepoint highly influenced by those ten minutes previously. The autoregression parameter (ϕ) was estimated at 0.876 (95 % HDI: 0.869, 0.883). There was no significant interaction between number of piling events and piling duration (estimate:1.00; 95 % HDI: 0.9998, 1.0006), nor was there a main effect of the number of piling events (estimateLBackspace: 0.998; 95% HDI: 0.995, 1.00), nor the duration of piling events (estimate: 0.9998; 95 % HDI: 0.9990, 1.0004).

#### Random intercept model

The level of CO_2_ was estimated at an average of 1,041 (95 % HDI: 779, 1303). There was no significant interaction between number of piling events and piling duration (estimate: 1.00; 95 % HDI: 0.9998, 1.002), nor was there a main effect of the number of piling events (estimate: 1.01; 95 % HDI: 0.981, 1.04) or piling duration (estimate: 1.00; 95 % HDI: 0.996, 1.01).

From a welfare perspective, the overall results for CO_2_ are encouraging, given that the levels detected were all below DEFRA guidance of 3,000ppm and that piling did not increase these. As with temperature, however, we cannot rule out that sensor placement may not be adequate for detecting chicken-level concentrations. Though levels experienced may be more concentrated at the piling location, concentrations of even 5,000ppm have been reported to have no obvious effects on chickens (Anderson et al., 1966). Ranges of higher concentrations of CO2 (> 7.5 %) are studied throughout the slaughter literature and have been shown to be aversive. The chronic effects of low levels of CO_2_ exposure found in other species (e.g. drowsiness and impaired cognition) have not been studied in chickens, as far as we are aware.

Secondary to investigating the association between piling and the laying shed environment, this study highlights the need to consider behavioral impacts when modelling a temporally fluctuating outcome. As piling can often be undetected in a flock, we could not use data from assumed non-piling flocks to inform baseline temperature and CO_2_. We therefore chose to model time in four ways and assess the fit to our data. Autoregressive models, one of our chosen options, are common in time series modelling but the model fit is not directly comparable to non-autoregressive models. When we estimated the effect of piling on temperature using the autoregressive model and the next best fitting non-autoregressive model, we found different results. It is therefore important to exercise caution in interpreting the result for temperature described above. Research on livestock housing needs robust baseline data, such as temperature, humidity, and gas across locations and time. Understanding how managed animal behavior interacts with housing has important implications for health, welfare, housing design, and greenhouse gas emissions. This study emphasizes the need for more research on behavior-environment interactions, with sensors at animal-level, and the potential of environmental monitoring to alert for problematic behaviors.

## Declaration of competing interest

The authors declare the following financial interests/personal relationships which may be considered as potential competing interests: Lucy Asher reports financial support was provided by Biotechnology and Biological Sciences Research Council. Helen Gray reports financial support was provided by Biotechnology and Biological Sciences Research Council. Jack O'Sullivan reports financial support was provided by FAI Farms Ltd. Lucy Asher reports equipment, drugs, or supplies was provided by FAI Farms Ltd. Lucy Asher reports equipment, drugs, or supplies was provided by The Lakes Free Range Egg Company. Lucy Asher reports a relationship with Biotechnology and Biological Sciences Research Council that includes: board membership. If there are other authors, they declare that they have no known competing financial interests or personal relationships that could have appeared to influence the work reported in this paper.
